# How advocacy coalitions in Sweden explain the policy gap between Swedish and EU eel fishery policies

**DOI:** 10.1007/s13280-024-02117-1

**Published:** 2024-12-20

**Authors:** Jens Nilsson, Annica Sandström

**Affiliations:** Department of Social Sciences, Technology and Arts, Luleå University of Technology, 971 87 Luleå, Sweden

**Keywords:** Advocacy coalitions, *Anguilla anguilla*, European eel, Management, Policy, Sweden

## Abstract

Our study explores governing of European eel in Sweden. The paper aims to analyze and tentatively explain the degree of policy coherence between different political levels and discuss implications for management. The study focuses on the Advocacy Coalition Framework and a qualitative methodology. Results show that EU and Swedish eel fishery policies are based on partly different beliefs about prioritized groups, problem descriptions, and policy preferences. Swedish policy is more considerate of fishery, attentive to the problems of hydropower, and hesitant toward fishery closures, than is the EU. These differences can be understood by the positions and power of the two advocacy coalitions competing for influence at the national level. National decisions align more with the coalition that includes fishery organizations, the Swedish Board of Agriculture, and coastal municipalities than with the beliefs of the coalition involving environmental—and sport fishing organizations and the Swedish Environmental Protection Agency.

**Supplementary Information:**

The online version contains supplementary material available at 10.1007/s13280-024-02117-1.

## Introduction

Although the critical situation of European eel stocks has been known about for years, and ambitious conservation goals have been adopted along the way, there are few signs of the stock recovering (Council Regulation No. 1100/2007; ICES 2023). Eel management is associated with several governance challenges related both to scientific uncertainties, such as limited knowledge about the species and its migration patterns, and to institutional uncertainties that stem from a complex multi-level governance structure (cf. Rittel and Webber 1973; Koppenjan and Klijn 2004; Dekker 2016). Efforts to protect the eel have been made at the European level, and in 2007 the European Union (EU) adopted a regulation to enhance the recovery of the eel stock. This regulation acknowledges the critical status of the European eel. It requires member states to act and implement necessary measures related to several areas, such as aquaculture, fishery, hydropower turbines, and restocking, to combat the negative trend (Council Regulation No. 1100/2007). While the actors involved in policymaking and management, as well as those participating in the public debates—e.g., non-governmental organizations (NGOs), industries, an researchers—seemingly support the overall goal to protect the European eel, there seems to be less consensus with regard to how (van Herten and Runhaar 2013; Garrido et al. 2020). The situation for the European eel is subject to intensive discussions among policymakers, interest organizations, industries, and researchers. This study explores the multi-level policy subsystem governing the European eel in Sweden, to increase our understanding of the present situation. We focus particularly on the role of advocacy coalitions, which are clusters of actors who hold similar beliefs about goals and solutions and take action to influence these (cf. Matti and Sandström 2013; Weible et al. 2020).

Eel management is a controversial issue in the Swedish context since conservation values and biological considerations, such as the ones emphasized by the EU regulation (Council Regulation No. 1100/2007), sometimes conflict with other values, particularly the historical Swedish cultural values associated with traditional eel fishery (cf. Stage 2015). There are different views on how much, by who, where, and when to fish eel. Previous research has suggested that these conflicting goals have not been appropriately handled in national politics and that the need for further deliberative political priorities has been neglected (cf. Svedäng and Gipperth 2012). Consequently, the trade-offs between values that are manifested by important decisions at the national level, such as the annual eel fishery, might or might not differ from the intentions stipulated at the European level. Differences between levels are not necessarily a problem, since we expect the EU legislation to be specified and adapted to national conditions on its way to implementation, but they can be if the two levels are governed by competing beliefs about goals and solutions. Policy coherence can be defined as congruence in goals and rules (Nilsson et al. 2012), and previous research has identified signs of deficient policy coherence between governance levels in the European system (Dekker 2016). However, we do not know whether, to what extent, and how the two policy levels (EU and Sweden) currently concur and diverge. These issues must be explored empirically to determine the presence of policy gaps.

Policymaking in multi-level governance systems is known to be challenging, and the presence of vertical policy incoherence (Nilsson et al. 2012; Söderberg 2016; Sandström et al. 2020)—e.g., when one policy level puts more emphasis on prioritizing environmental goals and the other focuses more on economic goals—is a complicating factor. We also know that EU legislation that does not conform with the member states’ institutional and regulatory traditions can be challenging to realize (Knill and Lenschow 1998; Hartlapp and Leiber 2010). The decisions of actors further down the policy chain, influenced by their understanding of policies, their networks, and resources, can also affect implementation (Sevä and Sandström 2017), and increase the gaps between higher-level policy and decision-making on lower levels (Giessen and Krott 2009; Sarvašová et al. 2013; Lundmark 2020). The above-discussed challenges of multi-level governance are likely also relevant for this case.

Thus, decisions influencing the future of the European eel are shaped by complex policy processes. Public policies are defined as decisions “of a government or an equivalent authority” (Weible 2014, p.4) that are “designed to achieve defined goals and present solutions to societal problems” (Knill and Tosun 2020, p. 13) and encompass “sets of value priorities and causal assumptions about how to realize them” (Sabatier 1988, p. 131). These fundamental positions expressed in, e.g., laws, regulations, and national programs, reflect the policy core beliefs, i.e., positions on value priorities, problems, and solutions, of the most powerful advocacy coalition (Sabatier and Jenkins-Smith 1999). Since the structure of advocacy coalitions—their policy core beliefs, resources, and power—may differ across levels, we can encounter policy incoherencies. Thus, to understand the policy dynamics and to be able to understand and explain the presence or lack of policy coherence between the EU and Sweden, we should identify the advocacy coalitions, explore their beliefs and strategies, and see how their positions are translated (or not translated) into decisions.

### Aim

Our study explores the multi-level governing of the European eel fishery in Sweden. The paper aims to analyze and tentatively explain the degree of policy coherence between levels and to discuss its implications for management. First, we conduct a policy analysis to identify and compare the policy core beliefs expressed in the EU policy with formulations expressed in Swedish policy documents. Second, we analyze the presence and positions of advocacy coalitions in the search for explanations for national decisions and the degree of coherence between the two levels. Third, and based on the above, we discuss the implications of the findings for management in the Swedish context.

The study will contribute with policy-relevant knowledge about whether and how Swedish eel policy differs from EU policy, and provide information about the structure and position of advocacy coalitions in the Swedish eel fishery context, shedding light on how and why we may encounter a policy gap across levels. The Swedish eel case illustrates the many governance challenges of managing endangered species in multi-level governance settings, with high scientific uncertainties, competing values, and strongly organized interest groups. Information about how to understand and navigate such settings can be useful for researchers, policymakers, and managers who are interested the governance of species and natural resources that are contested in complex institutional contexts.

### Theory

This study is based on ideas and concepts developed within the Advocacy Coalition Framework (ACF). The ACF was originally constructed to better understand and explain policymaking associated with societal problems characterized by goal conflicts, polarized opinions, and interactions across multiple levels of the political system (Sabatier and Jenkins-Smith 1999; Sabatier and Weible 2019). The framework has been frequently applied in studies of environmental policy processes in different contexts and in different parts of the world (cf. Sotirov and Memmler 2012; Nohrstedt and Olofsson 2016; Pierce et al. 2017).

The policy subsystem, which is delineated by the specific policy issue, the actors involved, and territorial boundaries, constitutes the basic unit of analysis (Sabatier and Jenkins-Smith 1999), and the advocacy coalitions are key entities in the framework, since the policy output is considered to reflect the beliefs of the dominant, i.e., most influential, coalition (Sabatier 1988; Weible et al. 2020). As mentioned in the introduction, these coalitions are composed of various actors, such as elected politicians and public officials with formal decision-making authority, but also representatives of interest organizations, industries, and even scientific institutions. Shared beliefs and more-or-less coordinated efforts to influence the process unite the actors within coalitions and separate them from other coexisting coalitions in the system^1^ (Weible et al. 2020). We can expect the power and interplay of coalitions in the policy subsystem to differ. The ACF distinguishes between adversarial, unitary, and cooperative subsystems, where the first type indicates two rival and internally coherent coalitions with little or no interaction in between; the second illustrates the presence of one dominating coalition; and the third includes some collaboration across coalitions (Weible 2008; Weible et al. 2020). We will identify the presence of advocacy coalitions in the Swedish eel fishery policy subsystem by analyzing the actors’ policy core beliefs.

The ACF differentiates between three different types of beliefs—deep core beliefs, policy core beliefs, and secondary aspects, according to their scope and stability (Weible and Sabatier 2007, following Sabatier 1998). Theory and empirical studies indicate that policy core beliefs, defined as relatively stable and fundamental positions on value priorities, problems, and solutions, are most critical for coalition formation (Sabatier and Jenkins-Smith 1999; Matti and Sandström 2013; Weible et al. 2020). These policy core beliefs can be further separated into normative and empirical policy core beliefs following the conceptualization of Sabatier and Jenkins-Smith (1999). Normative policy core beliefs refer to the orientation on basic value priorities and identification of groups of greatest concern for the policy. Applied to the context of the European eel, debates on where to draw the line on the scale between economic gains and conservation benefits, and whose interest to prioritize in the eel fishery context, belong to this category. The empirical policy core beliefs capture perceptions of policy problems and their solutions. These beliefs can be illustrated by different views on the urgency of the situation, e.g., how endangered the eel is and positions on what the primary causes of the problem are, e.g., to what extent the fishery and hydropower industry contribute to the problem. Different views regarding by whom and with what policy instruments the problems should be handled can also exist, e.g., supporting the central position of national public authorities and stronger regulations, or emphasizing the involvement of interest organizations and local and voluntary solutions. Finally, different policy core preferences, such as positions on the eel fishery being open, banned, or regulated, also belong to the category of empirical policy core beliefs (see Table 1).

**Table 1:** Questions to guide the empirical analysis of policy core beliefs in policy documents, consultation responses, and media

*Normative policy core beliefs*	
Orientation on basic value priorities	What is the primary goal of eel management and why is this goal more important than other goals?
Identification of groups of greatest concern	Whose interest is most important to consider and why is this so?
*Empirical policy core beliefs*	
Problem description	What are the major causes of the problems and how serious are they?
Proper distribution of authority	What actors, on what levels, should handle the problem?
Priority accorded to various policy instruments	What policy instruments should be used to handle the problem?
*Policy core preferences*	
Positions on core aspects of the policy	Should fishery be allowed, and if so, to what extent, when, and how?

We will analyze policy at different levels and identify the advocacy coalitions engaged in the process to understand policy dynamics and decisions in the Swedish eel fishery policy subsystem:

1. What fundamental policy core beliefs are evident in EU-level and national-level eel fishery-related policies, and are there any significant differences between the two?
2. What advocacy coalitions exist within the Swedish eel fishery policy subsystem, what are their policy core beliefs, and which coalition’s beliefs are reflected in national policy decisions?

## Materials and methods

This study is a qualitative content analysis of policy documents and statements in public consultation processes and media. The study deals with the Swedish eel fishery policy subsystem and focuses particularly on the interaction between the EU and national levels. Considering the 16 national environmental quality goals,^2^ the management of eel stocks is foremost subject to the goals: “A balanced marine environment and flourishing coastal areas and archipelagos,” “Flourishing lakes and streams,” “A rich diversity of plant and animal life,” and “A non-toxic environment” (Government bill 2004/05:150). Considering the legislative framework, the eel is foremost affected by the fishery laws (SFS 1993:787; FIFS 2004:36; FIFS 2004:37), the Swedish Environmental Code (SFS 1998:808), and past (yet still binding) court decisions about fish stocking made in association with permits for hydropower investments (National Fishing Agency 2008).

National policies that explicitly target the eel in Sweden are the results of EU policy and regulations. The eel regulation, mentioned in the introduction, is the most central (Council Regulation No. 1100/2007). The regulation requires member states to develop national management plans, and the Swedish management plan was introduced in 2008 and approved by the European Commission in 2009. The plan identifies management responses in four areas: 1) fishery, 2) hydropower, 3) restocking, and 4) monitoring activities (National Fishing Agency 2008).

More recent changes in EU fishery regulations have also significantly impacted national policy and management. The EU imposed a three-month temporary marine eel fishery closure in 2017. This decision was modified in 2019 to include all life stages of the species and further revised in 2022, imposing a six-month temporary marine eel fishery closure [COM (2017)645 final; COM (2022)559 final]. Swedish eel fishery is also restricted to commercial fishermen with a permit, and only allowed on the Swedish east coast and in a few inland waters during certain periods of the year (FIFS 2004:36). Recreational fishing of eel is, however, excepted from fishery prohibition when performed in some freshwater areas where hydropower dams prevent eels from migrating downstream (FIFS 2004:37). Based on the EU fishery regulation presented above, the Swedish Agency for Marine and Water Management (SwAM) makes yearly decisions about when fishery is allowed and when it is not. It is clear from the above that these decisions foremost affect the commercial fisheries.

To get an overview of the policy subsystem and determine which policy documents to include in the analysis, we searched for and scrutinized the laws, bills, and policies related to Swedish eel fishery. This selection was guided by the information provided on SwAM’s homepage (listing relevant policies and regulations),^3^ previous literature, and complemented with manual searches. We selected key documents according to the criteria that they a) govern eel fishery management in Sweden and b) comprise expressions of policymakers’ policy core beliefs to answer Question 1. Examples of documents that were excluded, since they do not fulfill the criteria, are law texts only stating jurisdiction but not beliefs (such as the fishery law, SFS 1993:787), as well as more general policy documents not explicitly directed at the eel (such as the Swedish Environmental Code, SFS 1998:808). These documents were, however, consulted for the case description presented herein.

For the European level, we include the EU regulation on establishing measures for the recovery of the stock of European eel (Council Regulation No. 1100/2007); the evaluation of national eel management plans [COM(2014)640 final]; and the two proposals from the Council of the European Union [COM(2017)645 final; COM(2022)559 final] concerning annual temporary marine eel fishery closures. For the national level, we analyzed the national eel management plan (National Fishing Agency 2008), the National Fishing Agency’s (changed in 2011 to SwAM) prescriptions on fishery (mainly FIFS 2004:36; 2004:37), and SwAM’s annual proposals (2018, 2019, 2020, 2021a, b, 2022, 2023) to revise the fishery law (FIFS 2004:36) with temporary marine eel fishery closure. These documents align with the above-presented criteria and provide a good basis for a comparative analysis of what policy core beliefs govern the EU level and national level.

All policy documents were uploaded in the qualitative data analysis software NVivo 14 (lumivero.com) and thematically coded (Williams and Moser 2019) based on the analytical framework rooted in the ACF, and are presented in Table 1. The first column of the table lists the policy core beliefs, and the second column presents the questions asked to capture these beliefs in the empirical material. The formulation of questions was adapted to the eel policy context but inspired by previous ACF research (e.g., Nwalie 2019; Sandström et al. 2021; Crawford and Weible 2024). Each belief category was given its own code in NVivo, each document was carefully read, and expressions of policy core beliefs coded (see examples of the coding in Appendix S1). The coded data were then aggregated and analyzed.

Before decisions about the annual temporary marine eel fishery closures are made, SwAM invites concerned actors, e.g., public authorities, scientific organizations, and NGOs to share their opinions on their proposals in written statements. We use these consultation responses, between 2018 and 2023, to a) identify key actors and b) map the structure of advocacy coalitions based on their beliefs (and answer Question 2). To this information, we add an analysis of by whom, and how, the eel issue has been debated in the media between 2008 and 2023. To determine which coalition’s beliefs are translated into decisions (Q2), we compare the policy core beliefs of the advocacy coalitions with the final decisions made by the national agency.

To identify advocacy coalitions, we analyzed the statements and arguments of each actor in the consultation process related to SwAM’s closure proposal and grouped them according to similarities in expressed beliefs (guided by Table 1). The same principle for coding beliefs and identifying coalitions was applied in the media analysis. The media archive Retriever (retriever.se) was used to identify opinion-based media articles related to eel management between the years 2008 and 2023. The initial search strings, based on the results of our initial overview of the policy subsystem mentioned above, were “eel AND fisher*” and “eel AND management.” After scrutinizing the results, we found that the last search string (management) could be excluded since it did not add any significant unique articles to the sample.

The choice of which articles to include in the media analysis was guided by the following criteria: a) Article expresses opinion on eel in relation to fishery, b) article is not a reproduction of the same information (for example, newspapers may re-report an opinion piece in another paper as a new article), c) article is not a rewrite of another article (for example, newspapers sometimes publish the same article with different titles), and d) article presents author and affiliation. A total of 134 articles fulfilled these criteria. We screened and marked each article as “included in the analysis,” “further reading necessary,” or “excluded from the analysis.” In the next step, the articles labeled as “further reading necessary” were reread and then labeled as either “included in the analysis” or “excluded from analysis.” This procedure resulted in a final selection of 54 articles. The articles were analyzed thematically in the same way as the consultation responses to SwAM’s proposal (guided by Table 1).

## Results

Table 2 presents the results of the analysis of policy core beliefs expressed at the EU and national levels.

**Table 2:** Policy core beliefs expressed in EU policy and Swedish policy

	EU	Sweden
*Normative policy core beliefs*		
What is the primary goal of eel management and why is this goal more important than other goals?	To protect and use European eel *(Anguilla anguilla)* sustainably in EU Community waters, coastal lagoons, estuaries, rivers, and adjacent inland waters. Reduction of anthropogenic eel mortality should be to as close to zero as possible (including fisheries) and aim to achieve at least 40 percent of the theoretically possible silver eel migration (as if no anthropogenic factors influence the stock)	To scale conservation activities (including restrictions on fisheries) for European eel *(Anguilla anguilla)* in such a way that the negative recruitment level is reversed. At least 90 percent of all European eel (silver eel) that potentially could be generated in Swedish waters should survive and contribute to reproduction
Whose interest is most important to consider and why is this so?	European eel (*Anguilla Anguilla)* is of primary concern	While the European eel (*Anguilla anguilla)* is of primary concern, management actions should not be to an extent that they challenge the livelihood of remaining commercial fishers on the Swedish east coast
*Empirical policy core beliefs*		
What are the major causes of the problems and how serious are they?	European eel population is below safe biological limits and decreasing at an alarming rate to historically low levels; fishery (and non-fishery-related anthropogenic) eel mortality is not sustainable	European eel population is in crisis. Several causes of this problem, the most prominent being fishery and hydropower-related mortality. Other causes are destruction of habitats, environmental toxins, predation, and (perhaps also) diseases/parasites
What actors, on what levels, should handle the problem?	Decisions should be taken as close as possible to eel exploitation areas. Member states’ authority important to enable management adjusted to regional and local conditions, but cooperation and action should also take place at all levels (EU community, national, regional, local); European Commission responsible for approving member states’ planned conservation actions	Public authorities at national and regional levels are primarily responsible for implementation, but coordination with neighboring countries with shared waters (especially Denmark) and other EU member states is important for implementing conservation efforts equally
What policy instruments should be used to handle the problem?	Regulative management plans to reduce anthropogenic eel mortality to as close to zero as possible, which includes reduction of commercial and recreational fishing, temporary marine eel fishery closures	Management plan includes reduction of commercial and recreational fishery. Temporary marine eel fishery closures have also been implemented (although the latter six-month reluctantly). Fishery and hydropower turbines result in most eel mortality and management efforts toward the latter should be prioritized, since fishery has already been regulated in several ways
*Policy core preferences*		
Should fishery be allowed, and if so, to what extent, when, and how?	Yes, but member states should adjust to sustain the most efficient implementation of the goal to reduce anthropogenic eel mortality to as close to zero as possible, including fishery restrictions, in their contexts	Yes. Sustainable commercial fishery should be allowed as long as it does not challenge the goal of reversing the negative recruitment level. No new licenses should be granted to fishers not already holding one

The comparison between levels shows several similarities. The EU regulation (Council Regulation No. 1100/2007) and the Swedish national eel management plan (National Fishing Agency 2008) express comparable beliefs in several regards. This was expected (but could not be assumed) since the Swedish management plan was developed from the EU regulation. A sense of urgency for eel population recovery is prominent, and both levels are guided by conservation goals, which affect fishery. The EU-level goals are to reduce all anthropogenic eel mortality to as close to zero as possible [COM(2022)559 final] and to ensure the survival of at least 40 percent of the theoretically possible silver eel migration (as if no anthropogenic factors affected the stock) (Council Regulation No. 1100/2007). The Swedish policy documents echo the EU 40 percent goal and include a national goal to ensure that at least 90 percent of all eels that could be generated in Swedish waters survive.

However, a comprehensive analysis of all policy documents included in the sample shows some differences in normative and empirical policy core beliefs. For the normative beliefs, when scrutinizing all policy documents over time, the EU-level policies express the goal of reducing all anthropogenic eel mortality to as close to zero as possible, as well as the urgent need to maximize the protection and recovery of eel stocks [COM(2022)559 final]. The Swedish policy documents keep the targets close to what is stated in the national management plan (that at least 90 percent of all eels that potentially could originate in Swedish waters should survive and contribute to reproduction) and refer to the goal of at least 40 percent of the possible silver eel migration that is expressed in the EU regulation (Council Regulation No. 1100/2007). In addition, national academic experts supporting SwAM reserved against leaning only on ICES advice, since these advice (according to the Swedish academic experts) lack possibility to evaluate goals for all anthropomorphic effects on eel mortality and do not comply fully with the EU regulation (SwAM 2021b). The normative beliefs on goals for eel management here differ between EU and national levels.

A significant difference concerns the identified group(s) of concern. The Swedish policy’s concern for the commercial eel fishery can be noted and contrasted with the beliefs expressed the EU level (Table 2). The arguments underpinning the Swedish government’s objection to extend the eel fishery closure period in 2022 is an illustrative example of this (Government Offices of Sweden 2022/23:FPM22).

Some differences in empirical core beliefs can also be noted in Table 2. The EU requires member states to increase non-fishery-related policy instruments, since these activities are still relatively unregulated, as well as to impose new instruments to constrain fishery further. A difference in emphasis can be noted when this view is compared with the beliefs expressed in Swedish policy (in particular, FIFS 2004:36). Fishery and hydropower turbines are highlighted as the main factors behind eel mortality in the national documents and followed by the argument that implemented fishery regulations have been successful in decreasing catches, while hydropower turbine regulations are insufficient. Thus, the measures related to hydropower mortality should be prioritized.

In line with the above, the Swedish government objected to the EU commission’s proposal to expand the temporary marine eel fishery closure from three to six months in 2022 [COM(2022)559 final] and suggested that other actions should be taken, e.g., in freshwater areas, to maintain a cautious and sustainable commercial eel fishery on the east coast (Government Offices of Sweden 2022/23:FPM22). Both past and present government ministers in Sweden have argued in parliament interpellations for the need for and tradition of a sustainable eel fishery (Swedish Parliament 2020, 2022). To summarize, the empirical analysis implies similarities but also differences, foremost related to prioritized groups of concern, problem perceptions, and policy core preferences, between the two levels.

Table 3 shows the grouping of actors into advocacy coalitions based on their responses to SwAM’s annual proposals (2018–2023) for temporary marine eel fishery closures. The “x” in the table marks whether one or more actors within the coalition were in favor of the suggested period (‘pro’), wanted it to be adjusted (‘with modification’), or were against it (‘against’). When interpreting the results, it is important to note that some of the actors that replied to the proposal were not grouped into either of the two coalitions. The reason for this was that these actors, all of them related to public authorities in Sweden, simply supported the suggested periods without further motivation (which makes belief mapping impossible), withdrew from making a statement since they felt that it was not within their authority to do so, or were asked to comment only on the procedure, rather than expressing views on the suggested periods (as in the case of the Swedish Council of Legislation). Even though some overlaps in responses between coalitions can be noted in Table 3, the analysis of policy core beliefs (presented below) verifies the presence of two internally coherent coalitions.

**Table 3:** Advocacy coalitions identified in consultation responses to annual proposals (2018–2023) for temporary marine eel fishery closures

Policy proposal on temporary marine eel fishery closures	Coalition A			Coalition B			Agency decision
	Commercial fishery organizations, fishery NGOs, eel fishery and culture NGOs, the Swedish Board of Agriculture, coastal municipalities in southern Sweden (Höganäs, Karlshamn, Karlskrona, Landskrona, Ronneby & Sölvesborg), eel farming company, private eel fishers			Environmental NGOs, sport fishing and management association, research center (Stockholm University Baltic Sea Centre), Swedish Environmental Protection Agency, County Administrative Board of Skåne			
	Pro	With modification	Against	Pro	With modification	Against	
2018 (November 1, 2018, to January 31, 2019)	X		X	X	X**		Proposed eel fishery closure was applied
2019 (November 1, 2019, to January 31, 2020)	X	X*			X**	X	Proposed eel fishery closure was applied
2020 (November 1, 2020, to January 31, 2021)	X	X*^/^***			X**	X	Proposed eel fishery closure was applied
2021(November 1, 2021, to January 31, 2022)	X	X*^/^***	X		X**	X	Proposed eel fishery closure was applied
2022 (October 1, 2022, to December 31, 2022)	X	X*^/^***	X	X	X**	X	Proposed eel fishery closure was applied
2023 (October 1, 2023, to March 31, 2024)	X				X**	X	Proposed eel fishery closure was applied

*Argues for later fishing closure and/or removal of fishing closure periods

**Argues for earlier fishing closure and/or total stop to eel fishery

***Argues for coordination with Denmark

Coalition A consists of commercial fishery organizations, fishery NGOs, eel fishery and cultural heritage NGOs, the Swedish Board of Agriculture, coastal municipalities in southern Sweden (Höganäs, Karlshamn, Karlskrona, Landskrona, Ronneby, and Sölvesborg), an eel farming company, and private eel fishers. Most actors in Coalition A responded to most of, or all, the proposals between 2018 and 2023. The municipalities only responded to two of them, the eel farming company only one, and private eel fishers responded occasionally. The common denominator for this coalition is that they are in favor of the proposed closure periods or wish to move them to a later period when eel fishery is less active. Some of them also argue for a removal of the temporary marine eel fishery closures. The only actor who is consequently following the proposals is the Swedish Board of Agriculture. Several actors within Coalition A express criticism of SwAM; they question the introduction of further regulations on Swedish eel fishery and stress the lack of hydropower regulations and coordination with Denmark. The decision not to provide new fishery licenses is also questioned, and arguments for removal of this regulation, to ensure a generational change in eel fishery, occur in several statements. However, all coalition actors supported the suggested temporary marine eel fishery closure in 2023 (which was heavily criticized by Coalition B and is further discussed below).

Coalition A also emphasizes that values other than the conservation of the European eel need to be taken into consideration in policymaking—particularly those related to the cultural value of eel fishery in Sweden. Furthermore, the most unifying belief within the coalition is the common problem perception. Actors in Coalition A view mortality from hydropower turbines as the most significant challenge in preserving the species and express the need to take action to reduce this mortality as the most urgent policy priority. When making this argument, several actors within the coalition also emphasize that fishery has already been significantly reduced and is not in need of further restrictions, since additional measures would challenge the sustainability of commercial fishery and the cultural heritage related to eel fishery and consumption.

Coalition B consists of environmental NGOs, a sport fishing and management association, a research center (Stockholm University Baltic Sea Centre), the Swedish Environmental Protection Agency, and the County Administrative Board of Skåne. In Coalition B, actors either want to move the proposed temporary marine eel fishery closure to earlier in the season, when eel fisheries are more active, or suggest a total stop of the fishery. The trend is generally persistent over time. The County Administrative Board of Skåne is the only actor to express support for some of the suggested periods (in 2018 and 2022) but base their positions on the need for better coordination between Swedish and Danish regulations (to align the periods between countries).

Coalition B perceives the as eel as severely threatened and the primary unit of concern for policymaking. The problem description is characterized by a sense of urgency to support the species’ survival, which is challenged in several ways, including by fishery and mortality due to hydropower turbines. This coalition perceives the eel fishing periods suggested by SwAM as problematic and argues that the national agency’s interpretation of scientific advice from the EU and the International Council for the Exploration of the Seas (ICES) is incorrect. The policy instruments suggested by this coalition imply that all possible measures to reduce anthropomorphic eel mortality to as close to zero as possible are needed, encompassing temporary marine eel fishery closures when most fishing activities occur (or a total stop) in combination with stricter regulations for hydropower and other causes of anthropomorphic eel mortality. The six-month period suggested in the last proposal (SwAM 2023) was heavily criticized by this coalition, with the argument that the chosen period included time when no commercial eel fishing would be taking place so, consequently, it would have little impact on eel mortality.

Based on the above, we see that the two coalitions express different beliefs about value priorities, problem perceptions, and priority according to various policy instruments and policy core preferences. It should, however, be acknowledged that several similarities between the two groups can also be noted. Actors across the coalitions emphasize a concern for the eel population and agree with the need to take management actions for their conservation.

The presence and characteristics of the two coalitions are verified in the media analysis. However, some differences in the actors’ participation in the media debate can be noted. The political party representatives are active in the media but the voices from public authorities are missing. We can see that political parties to the right of the political spectrum are aligned with Coalition A and two political parties to the left are aligned with Coalition B. Table 4 illustrates the coalitions, their actors, and their expressed beliefs, as found in the media analysis.

**Table 4:** Advocacy coalitions related to eel fishery identified in the media analysis

	Coalition A	Coalition B
*Coalition actors*		
	Commercial fishery organizations, fishery NGOs, eel fishery and culture NGOs, private eel fishers, political parties (the Liberal Party, the Moderate Party, the Center Party, the Christian Democrats, the Swedish Democrats)	Environmental NGOs, sport fishing and management association, researchers, political parties (the Green Party, the Social Democratic Party, the Left Party)
*Normative policy core beliefs*		
What is the primary goal of eel management and why is this goal more important than other goals?	To preserve and protect the European eel (*Anguilla anguilla);* to preserve and protect cultural heritage related to eel fishery and eel as a traditional delicacy	To preserve and protect the European eel (*Anguilla anguilla)*
Whose interest is most important to consider and why is this so?	European eel (*Anguilla anguilla)*, eel fishers, local, regional, and national culture, consumers	European eel (*Anguilla anguilla)*
*Empirical policy core beliefs*		
What are the major causes of the problems and how serious are they?	The European eel species is critically endangered. Fishery has already been heavily regulated, so main threats are hydropower turbine- and predator-related eel mortality. Cultural heritage and livelihood are threatened because of strict fishery regulations	The European eel species is critically endangered. Several causes in the Swedish context, fishery and hydropower turbines being the greatest
What actors, on what levels, should handle the problem?	Not mentioned generally, although some articles direct criticism toward the EU level for not putting pressure on other member states, as well as for the regulations being too strict on fishery	Not mentioned generally
What policy instruments should be used to handle the problem?	Hydropower plants and turbines should be further regulated to prevent eel mortality. Management also needs to take action against eel predators, such as the cormorant (Phalacrocoracidae)	All anthropogenic eel mortality should be reduced and further regulated, including fishery and hydropower
*Policy core preferences*		
Should fishery be allowed, and if so, to what extent, when, and how?	Sustainable fishery of eel population should be allowed and would benefit both the eel population and the consumers	Fishery greatest cause of eel mortality and as such, all fishery of eel should stop

Most media statements in the sample are expressed by a single organization or person, but there are some instances of coordinated effort between actors. For Coalition A, we find joint articles by the fishers, eel fishery and cultural heritage NGOs, as well as joint statements by political parties (the Liberal, Moderate and Center parties, and the Christian Democrats). For Coalition B, there are several instances of the environmental NGOs writing joint statements together with the sport fishing and management association.

The media analysis also adds some other interesting insights. Both advocacy coalitions are to some extent dissatisfied with Swedish eel management, but for different reasons—Coalition A for too strict fishery regulation (in relation to other regulations, including for hydropower) and Coalition B for allowing unjustified commercial fishery and a general lack of measures. When comparing the positions and argumentation of the two advocacy coalitions with the final decision made by SwAM (see right-hand column in Table 3), we see how the positions of authority align more with Coalition A than with Coalition B.

## Discussion and conclusions

What fundamental policy core beliefs are evident in EU-level and national-level policies, and are there any significant differences between the two (RQ1)? The empirical results imply both coherencies and incoherencies between the two levels. Both EU and Swedish policies emphasize the importance of protecting the European eel, acknowledge the threats to this species, and share a sense of urgency in developing more sustainable management practices related to fishery. This said, we also note how the normative foundations expressed in Swedish policy differ from the EU, primarily by putting greater emphasis on the commercial fishery as a group in need of consideration and objecting against further restrictions for this group.

We also note differences in the perceptions of the fishery as a problem and increased fishery regulations as a solution. Fishery and hydropower are identified as the main causes of eel mortality in the Swedish context, but the latter is expressed as the one most urgently in need of further regulations. The EU policy, however, acknowledges that more needs to be done also to reduce fishery. The two levels differ in beliefs about how and to what extent fishery should be allowed. We note that the EU represents a stricter position than Sweden does on this matter.

These results illustrate challenges related to environmental multi-level governance systems in general (Sevä and Sandström 2017; Eberl et al. 2021) and are in line with previous scientific studies on the governance of European eel management (Svedäng and Gipperth 2012; Dekker 2016). The differences found in the policy analysis can contribute to our understanding of the current and future interplay between EU ambitions and national-level management decisions. Implementation problems due to differences in goals between the EU and member states have been discussed in studies about EU climate change policies (Eberl et al. 2021), biodiversity policy (Sotirov and Storch 2018), and forest policy (Beland Lindahl et al. 2017). The role played by public authorities (i.e., SwAM in this case) in multi-level governance systems can also be problematic since they need to balance multiple and sometimes competing goals (from the EU, national government, and stakeholders) before making their decisions (cf. Bennett et al. 2022, in their study of multi-level governance relating to wildlife management). Add to this the scientific uncertainties related to the species (Dekker 2016), manifested in different views between ICES and the national academic advisors in goal perceivement and EU regulation alignment, which further complicates the ability of having policy coherence between levels. The role of science can, if the scientific field is unified in its views, enable alignment between levels by imposing strong recommendations. When not, scientific debates may instead fragmentize, which is a likely outcome when facing a policy issue surround.

What advocacy coalitions exist within the Swedish eel fishery policy subsystem, what are their core policy beliefs, and which coalition’s beliefs are reflected in national policy decisions (RQ2)? Two coalitions were found in the Swedish eel fishery policy subsystem. Both coalitions (A and B) emphasized the value of the European eel and the need for its protection. Coalition A also shows great concern for commercial eel fisheries, emphasizing the cultural value of eel fishery, and eel as a food delicacy. The most prominent differences between the two coalitions concern the problem descriptions. Coalition A states that commercial eel fishery is sustainable and that the threat to European eel survival lies in hydropower turbines and in predators, whereas Coalition B argues for all anthropogenic mortality to be reduced to as close to zero as possible (including fishery). The view on the key problem is consequently mirrored in the priority; these two coalitions give to different policy instruments as well as in their views on fishery regulations.

Both coalitions express dissatisfaction with current Swedish eel management. Still, the empirical analysis shows that Coalition A, composed of different fishery organizations, the Swedish Board of Agriculture, and coastal municipalities in the south of Sweden, constitutes the dominant coalition as their policy core beliefs and preferences align with the orientation of decisions made by SwAM. This is especially evident in relation to the extended temporary marine eel fishery closure (from three to six months) suggested in 2023. This proposal was a particular focus of debate, and the national government initially opposed the extension. The process ended with SwAM proposing a period that covered a period when little or no eel fishing took place. The decision was supported by Coalition A but heavily criticized by Coalition B. Coalition B, including environmental and sport fishing organizations, the Swedish Environmental Protection Agency, and the research center, is thus the opposing and less influential coalition in the Swedish eel policy subsystem. The advocacy coalition structure can tentatively explain why there are differences between national and EU policies (cf. Sabatier and Jenkins-Smith 1999, proposing that the winning coalition’s beliefs are reflected in policy). The results of our analysis show how power differences between two rival coalitions are manifested in their influence, or lack of influence, on key national decisions. The coalitions found in this study resemble advocacy coalitions in other adjacent policy areas, e.g., marine protection (Sandström et al. 2020), wildlife (Matti and Sandström 2013; Lundmark et al. 2018), and forests (Sotirov et al. 2021), and we encourage future studies to adopt comparative approaches to learn more about influence and the exertion of power, and polarization, in different environmental and natural resource policy subsystems.

With this knowledge, we can better understand the policy dynamics in the subsystem, the priorities made in national eel fishery policy and, also, consider the likelihood of policy being changed in the near future. Policy changes normally occur when coalitions’ beliefs, strategies, and/or resources are modified through learning, by influence of external events, e.g., ecosystem changes with profound impact on the eel population, rapid shifts in public concerns, or institutional changes in adjacent policy areas altering the power relationships (Sabatier and Jenkins-Smith 1999; Nohrstedt 2022). Given the strong dominance of Coalition A policy in the Swedish system, and lack of clear and unified scientific advice about what actions to take, we suggest that stability, rather than change, is expected unless disruptive events take place or more assertive enforcement of EU policy is imposed from the above.

Other unanswered questions in need of further investigation relate to the presence and characteristics of advocacy coalitions on the EU level, and the degree of policy coherence between different policy sectors (e.g., fishery and biodiversity) on the EU level. We do not know how and to what extent the goals and beliefs expressed in other fishery policies align with the ones expressed in eel policies. When different priorities are made in different policy sectors at higher policy levels, this results in institutional complexities and different interpretations at the national level (as described in the discussion above). The identified policy gap between the EU and Sweden illustrates the fact that goal conflicts and difficult trade-offs between competing values become more evident further down the policy chain. Thus, it would be interesting to know how both horizontal policy coherence across policy sectors and vertical policy coherence between levels play out in all levels of the policy subsystem governing the European eel.

## Supplementary Information

Below is the link to the electronic supplementary material.

Supplementary file1 (PDF 75 kb)

## Data Availability

All policy documents used for analysis are publicly available. European Union policy documents can be downloaded from the European Union homepage (https://eur-lex.europa.eu/homepage.html). Swedish policy documents can be downloaded from the Swedish Agency for Marine and Water Management homepage (https://www.havochvatten.se), the Swedish Parliament homepage for document and laws (https://www.riksdagen.se/sv/dokument-och-lagar), the Swedish Government homepage for documents (https://www.regeringen.se/dokument-och-publikationer), and by request from Swedish Agency for Marine and Water Management (annual proposals 2018–2023 for temporary marine eel fishery closures with actor responses).

## Footnotes

1 Shared policy beliefs are often used as a proxy for coordination when delineating these coalitions in empirical research (cf. Weible and Sabatier 2005; Ingold 2011).

2 The 16 national environmental quality goals characterize the environmental quality Sweden is aiming to achieve. For their specifications, see Government bill 2004/05:150.

3 www.havochvatten.se.
