# Publisher Correction: How advocacy coalitions in Sweden explain the policy gap between Swedish and EU eel fishery policies

**DOI:** 10.1007/s13280-025-02130-y

**Published:** 2025-01-25

**Authors:** Jens Nilsson, Annica Sandström

**Affiliations:** Department of Social Sciences, Technology and Arts, Luleå University of Technology, 971 87 Luleå, Sweden

**Correction to: Ambio** 10.1007/s13280-024-02117-1

In the original published article, the statement in the Funding section was incorrectly given as ‘Open access funding provided by Lulea University of Technology. L.S. contribution has been supported by the Spanish Ministry of Universities through Magarita Salas fellowship. N.F. contribution has been supported by MUST project, funded by the Strategic Research Council (SRC) established within the Research Council of Finland (Grant No. 358365, WP3 Grant Number 358368) and Digital Waters Flagship, funded by the Research Council of Finland under the Finnish Flagship Programme (Grant No. 359247).’ and should have read ‘Open access funding provided by Luleå University of Technology.’

The original article has been corrected.

