# Supplementary material for: How advocacy coalitions in Sweden explain the policy gap between Swedish and EU eel fishery policies
Source: Ambio. 2024 Dec 20;54(5):899–911. doi: 10.1007/s13280-024-02117-1 (PMC11965073; doi:10.1007/s13280-024-02117-1)
Supplement: Supplementary file 1 — Supplementary file1 (PDF 75 kb) [file 13280_2024_2117_MOESM1_ESM.pdf]

Ambio
Supplementary Information
This supplementary information has not been peer reviewed.

Title: How Advocacy Coalitions in Sweden Explain the Policy Gap Between Swedish and EU Eel-Fishery Policies

Authors: Jens Nilsson and Annica Sandström

Appendix S1. Examples of coding

**Quote**	**Source**	**Coded belief**	**Argument for coded belief**
"By maintaining a restricted eel fishery, the knowledge of the eel is preserved as cultural heritage. Should eel fishing cease, interest in eels will disappear."	Consultation response from NGO to annual temporary marine fishery closure (translated from Swedish).	Normative Policy Core Belief: Orientation on basic value priorities.	**Primary goal** is to maintain a restricted fishery, and this is more **important than other goals**, since it preserves knowledge of eel as cultural heritage and without it interest in eels will disappear.
"In other words, the situation for the European eel (*Anguilla anguilla*) is considerably more serious than the management authority wants to claim. When the stock reaches such a low density as is now the case throughout its range, effects on its capacity to recover can be significantly debilitating"	Consultation response from research center to annual temporary marine fishery closure (translated from Swedish).	Normative Policy Core Belief: Identification of groups of greatest concern.	The **interest most important to consider** is the species (*Anguilla Anguilla*), **since** the situation is serious and the low density of stock affects its capacity to recover negatively.
"The latest scientific advice from the International Council for the Exploration of the Sea (ICES) concerning European eel is that the stock is outside safe biological limits and that current fisheries are not sustainable."	COUNCIL REGULATION (EC) No 1100/2007.	Empirical policy core beliefs: Problem description.	Quote describes what the **problem** is (European eel outside safe biological limits) and connects this with one of the **causes** (current fisheries are not sustainable).
"Several authorities are responsible for the implementation of the eel management plan" (goes on to list responsible authorities, mostly on national level, and their specific responsibilities)	Swedish national eel management plan.	Empirical policy core beliefs: Proper distribution of authority.	Public **actors** mostly authorities on **national level** (for example fishery and energy authorities), but also on **regional level** (county administration boards) as they are responsible for handling the implementation of the plan.
"But in this context, we environmental organizations also want to highlight how important it is that power plants that produce very little electricity and are located in biologically important waterways are actually decommissioned."	Media debate article authored by environmental NGOs and sport fishing and management association (translated from Swedish).	Empirical policy core beliefs: Priority accorded to various policy instruments.	Quote describes what **policy instrument** (regulative in form of decommissioning of small hydropower plants) should be **prioritized** over another (keeping these plants that do not produce enough energy) and **handles the problem** (of safer fish passages and ecosystems in biologically important waters).
"Small-scale fishing increases the number of jobs in Blekinge, culture is preserved, and the unique archipelago environment is developed. Generational shifts must be facilitated and made possible through the transfer of fishing licenses, to give the industry confidence for the future."	Media debate article authored by politician from the Swedish Democrats (translated from Swedish).	Policy core preference: Positions on core aspects of the policy.	Fishery **should be allowed** at a small scale and licenses **should be** renewed to younger generation ensure the continuation of eel fishery.
